# Alcohol, Coffee, and Milk Intake in Relation to Epilepsy Risk

**DOI:** 10.3390/nu14061153

**Published:** 2022-03-09

**Authors:** Zhizhong Zhang, Mengmeng Wang, Shuai Yuan, Xinfeng Liu

**Affiliations:** 1 Department of Neurology, Jinling Hospital, Medical School of Nanjing University, Nanjing 210002, China; zhizhongn@126.com; 2 Department of Neurology, The First People’s Hospital of Changzhou, The Third Affiliated Hospital of Soochow University, Changzhou 213004, China; w935017050@163.com; 3 Unit of Cardiovascular and Nutritional Epidemiology, Institute of Environmental Medicine, Karolinska Institutet, SE-171 77 Stockholm, Sweden; shuai.yuan@ki.se

**Keywords:** alcohol, coffee, milk, epilepsy, Mendelian randomization

## Abstract

Alcohol, coffee and milk intakes have been explored in relation to epilepsy risk in observational studies; however, the results were not consistent. We performed a Mendelian randomisation (MR) study to evaluate the causality of these relationships. Genetic variants associated with alcohol, coffee and milk intake were adopted as instrumental variables. We obtained the summary data of epilepsy from the International League Against Epilepsy (ILAE) Consortium (15,212 cases and 29,677 controls) and FinnGen consortium (4588 cases and 144,780 controls). Genetically predicted alcohol intake was associated with a higher risk of epilepsy in the ILAE Consortium (odds ratio (OR): 1.22, 95% confidence intervals (CI): 1.02–1.45). The association in the FinnGen consortium remained consistent in direction. Combined analysis of ILAE and FinnGen databases further indicated that genetically predicted alcohol intake was associated with a higher risk of epilepsy (OR = 1.24; 95% CI, 1.06–1.47, *p* = 0.009). Genetically predicted coffee intake was not related to epilepsy risk, while higher genetically predicted milk intake was related to a lower risk of epilepsy (OR = 0.957; 95% CI, 0.917–0.999, *p* = 0.044). Our results suggest a detrimental effect of alcohol intake on the risk of epilepsy, while milk intake might be associated with a decreased risk of epilepsy.

## 1. Introduction

It is estimated that epilepsy affects over 70 million people worldwide [1], which brings a huge economic and social burden. Previous studies have been reported regarding the association between alcohol intake and epilepsy risk. However, these results are not consistent [2,3,4]. The effect of other modifiable lifestyle behaviors, such as coffee and milk intake, in epilepsy, has been scarcely explored [5,6]. Most notably, these results are at risk of reverse causation and confounding bias. Therefore, successful interventions targeting modifiable lifestyles to prevent epilepsy development are of great importance to lowering the disease burden. Moreover, alcohol, coffee and milk intake are changeable lifestyle behaviors; determining their causal relationships with epilepsy could reveal the potential pathogenesis of epilepsy and are of great significance for formulating efficient prevention measures. 

Mendelian randomization (MR) adopts genetic variance as an instrumental variable to mimic the effect of environmental exposures, such as alcohol, coffee and milk intake, to explore causality [7]. Since the single nucleotide polymorphisms (SNPs) one is born with are constant through a lifetime, MR could decrease reverse causation bias, suggesting that the outcome (epilepsy) could not modify a person’s genetic predisposition for the exposure. Moreover, SNPs are generally unrelated to other confounders, since SNPs are randomly assorted during conception. Therefore, we performed an MR study to evaluate the associations of alcohol, coffee, and milk intake with epilepsy risk. 

## 2. Methods 

### 2.1. SNP Selection

Instrumental variables of alcohol intake were obtained from the GSCAN (941,280 Europeans) [8], which identified 99 alcohol intake related-SNPs (drinks per week, *p* < 5 × 10^−8^). Moreover, the 99 alcohol intake related-SNPs were clumped for independence (r^2^ < 0.01; region size, 10 Mb) based on the Europeans data from the 1000 Genomes Project [9]. As a result, 84 independent SNPs were adopted as instrumental variables for alcohol intake (Supplementary Table S1).

Genetic instruments for coffee intake were selected from a large GWAS (375,833 Europeans) [10], which identified fifteen coffee intake related-variants (*p* < 5 × 10^−8^). These coffee intake related-variants were also clumped for independence (r^2^ < 0.01; region size, 10 Mb) [7]. As a result, 12 independent SNPs were adopted (Supplementary Table S2).

For milk intake, rs4988235 was adopted as an instrumental variable. SNP rs4988235 is strongly related to milk intake in European individuals, and an additional T-allele of rs4988235 was associated with 0.58 (95% CI: 0.49–0.68) glasses/week increase in milk intake among a Danish cohort (*p* = 9 × 10^−36^) [11]. 

When these instrumental variables for alcohol, coffee and milk intake were not available in the epilepsy dataset, proxy SNPs in linkage disequilibrium (LD, r^2^ ≥ 0.8) were adopted through LDlink (https://ldlink.nci.nih.gov/, accessed on 1 June 2021).

### 2.2. Outcome Data Sources

Genetic results for overall epilepsy were obtained from a large genome-wide analysis in the International League Against Epilepsy (ILAE) consortium (15,212 cases and 29,677 controls, Table 1) [12]. Details on cohorts and phenotype definition, study design, genotyping and quality control were described in the original study [12]. For each variant, the beta value and corresponding standard error were calculated with the approach described by Zhu et al. [13].

Moreover, we additionally adopted data from the FinnGen consortium (http://r4.finngen.fi/, accessed on 1 June 2021) to further validate the results for these exposures and epilepsy. The FinnGen Data Freeze 4 contains 4588 epilepsy cases and 144,780 controls (Table 1). 

Our study was based on publicly available data only. Ethical approval for each of the GWASs could be found in the original publications.

### 2.3. Statistical Analyses

We applied the random-effects inverse-variance weighted (IVW) approach as the main analysis. Results from the ILAE and FinnGen consortium were combined with the fixed-effects meta-analysis. We adopted the weighted median as sensitivity analysis [14]. We also conducted MR-Egger regression to evaluate the directional pleiotropy [14]. Finally, the MR-PRESSO method was applied to identify potential outliers [14].

For alcohol drinking, the odds ratios (ORs) of epilepsy were scaled to a one standard deviation increase in log-transformed drinks per week. For coffee, the ORs of epilepsy were scaled to per 50% increase in coffee intake. For milk, the OR of epilepsy was calculated per additional T-allele of rs4988235. Association with a *p-*value less than 0.017 (0.05/3 exposures) was considered as significant, and an association with a *p-*value between 0.017 and 0.05 was considered as suggestive evidence of association. We used MendelianRandomization [15] and MR-PRESSO [16] packages in R software to conduct the analyses.

## 3. Results

### 3.1. Selection of Genetic Variants 

Of the 84 independent SNPs for alcohol intake, 24 SNPs were unavailable in the ILAE database. Suitable proxy SNP (r^2^ ≥ 0.8 with the specified SNP) was available for 4 SNPs. As a result, 64 SNPs were available in the ILAE database. Moreover, the MR-PRESSO method identified two potential outliers (rs62044525 and rs10506274), which were excluded from subsequent analyses. Therefore, 62 SNPs were used in the ILAE database. In addition, 4 of the 84 SNPs were unavailable in the FinnGen consortium database. Suitable proxy SNP (r^2^ ≥ 0.8) was available for 3 SNPs; therefore, 83 SNPs were used in the FinnGen consortium database.

Of the 12 independent SNPs for coffee intake, 7 SNPs were unavailable in the ILAE database. Suitable proxy SNP (r^2^ ≥ 0.8 with the specified SNP) was available for 2 SNPs. As a result, 7 SNPs were available in the ILAE database. All the 12 SNPs were available in the FinnGen consortium database. For milk intake, rs4988235 was available in the FinnGen consortium. However, this genetic variant was unavailable in the ILAE database, and no suitable proxy variant was available at an LD r^2^ ≥ 0.8. Thus, the association between milk intake and epilepsy was only analyzed in the FinnGen consortium.

### 3.2. MR Analysis 

**Figure 1:**
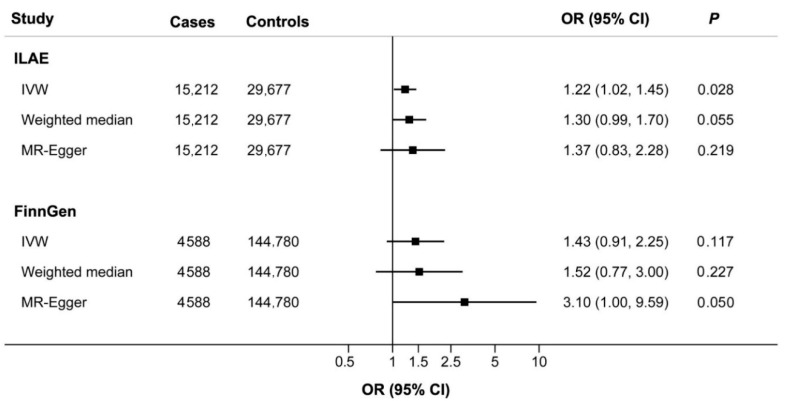
Associations of genetically predicted alcohol intake with epilepsy in the International League Against Epilepsy (ILAE) and FinnGen consortiums.

Overall, genetically predicted alcohol consumption was associated with a higher risk of epilepsy in the ILAE consortium (OR, 1.22; 95% CI 1.02–1.45, *p* = 0.028; Figure 1). Similar trends were observed in the weighted median method, although with wider CIs (OR = 1.30; 95% CI, 0.99–1.70, *p* = 0.055). In addition, the MR-Egger analyses indicated no evidence of directional pleiotropy (*p* > 0.05).

**Figure 2:**
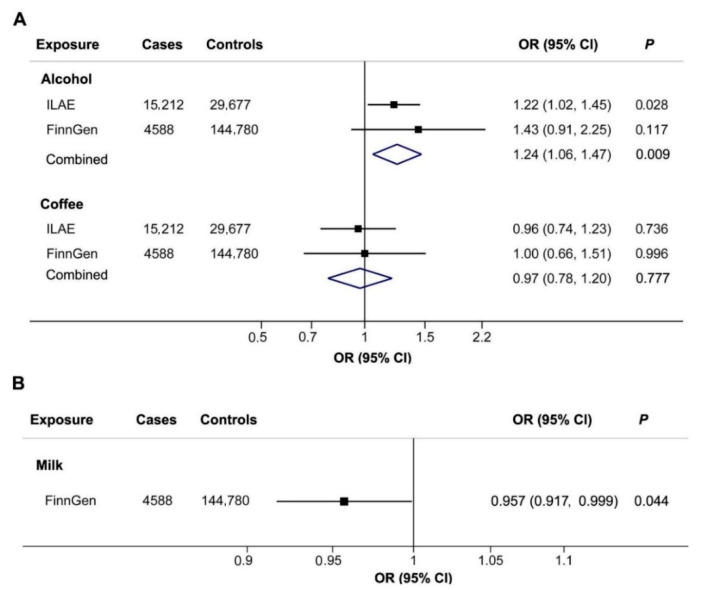
(**A**) Associations of genetically predicted alcohol and coffee intake with epilepsy in ILAE, in FinnGen, and combined analyses of both samples. (**B**) Association of genetically predicted milk intake with epilepsy in FinnGen.

The association between alcohol intake and epilepsy was further explored in the FinnGen consortium. As a result, an estimate is also directionally consistent (OR = 1.43; 95% CI, 0.91–2.25, *p* = 0.117, Figure 1). Combined analysis of ILAE and FinnGen databases further indicated that alcohol intake was associated with a higher risk of epilepsy (OR = 1.24; 95% CI, 1.06–1.47, *p* = 0.009, Figure 2A).

Genetically predicted coffee consumption was not related to epilepsy risk (OR: 0.96; 95% CI: 0.74–1.23; *p* = 0.736 in ILAE consortium; OR: 1.00; 95% CI: 0.66–1.51; *p* = 0.996 in FinnGen consortium, Figure 2A).

Genetically predicted milk intake showed a suggestive association with a lower risk of epilepsy in the FinnGen consortium. The OR was 0.957 (0.917–0.999, *p* = 0.044, Figure 2B) for each additional milk intake increasing allele (T).

## 4. Discussion

In our study, we explored the causal association between alcohol, coffee and milk intake and epilepsy risk using the MR design. We found that genetically predicted alcohol intake was associated with a higher risk of epilepsy, while genetically predicted milk intake showed a suggestive association with a lower risk of epilepsy in the FinnGen consortium.

A recent MR study was also suggestive of a detrimental effect of genetically predicted alcohol consumption on the risk of epilepsy (OR, 1.54; 95% CI, 0.99–2.41; *p* = 0.058) [17]. The insignificant association in this previous study is likely to be caused by an inadequate power. A previous observational study showed that alcohol intake was associated with a higher risk of epilepsy in a dose-response manner [3]. Karhunen et al. found that chronic alcohol intake could damage the cerebellum and lead to a significant loss of Purkinje cells [18]. Moreover, people with alcohol dependence have a higher incidence of head trauma and brain injury due to traffic accidents, falls, or attacks [19]. Epilepsy is a severe nervous system disorder worldwide. Thus, it is very important to formulate efficient prevention measures for epilepsy. According to the result of our MR study, alcohol intake should be limited for those at high risk of epilepsy.

Very few investigations have reported the association between coffee/caffeine intake and epilepsy risk [4]. A previous study showed that long-term caffeine intake was not related to the risk of epilepsy [4]. Our results were consistent with the conclusion from the previous study [4]. 

Evidence on the association between milk intake and epilepsy is also limited. The potential protective effect of milk on epilepsy may be mediated through calcium and vitamin D. In a Portuguese epilepsy cohort, more than half of the epilepsy patients showed vitamin D deficiency [20]. In a south Indian study, the calcium intake of epilepsy patients was also far below the recommended level [21]. In addition to calcium and vitamin D, other nutrients in milk may also have an effect on epilepsy development, which needs further research.

### Strengths and Limitations

Compared with conventional observational studies, the present MR study adopted summary data from GWASs with large sample sizes, which enabled us to draw clear conclusions and establish a causal relationship between these exposures and epilepsy. In addition, sensitivity analyses yielded similar results, which validate the robustness of the results. Moreover, we conducted the MR analyses in two large databases (the ILAE and FinnGen consortiums) and obtained consistent results, which further confirmed the association. 

Our study also has some shortcomings. First, we cannot evaluate the nonlinear association between these exposures and the risk of epilepsy, owing to the linear effect assumption of the MR analyses. Second, our study is mainly based on Europeans. Our study may not have the same results in other populations with equal or greater intakes of alcohol, coffee or milk. Third, the effect of milk intake on epilepsy was only available in the FinnGen consortium. Thus, whether milk intake influences the risk of epilepsy warrants further research. Fourth, the associations of other diet factors with the risk of epilepsy were not investigated in our study, which is worth further study.

## 5. Conclusions

The present study provides evidence that a genetically predicted higher alcohol consumption was associated with an increased epilepsy risk. Reducing alcohol intake should be regarded as an important prevention strategy for epilepsy. 

## Figures and Tables

**Table 1:** Studies and datasets adopted in the MR analyses.

Datasets	Ancestry	Sample Size	Consortium
Outcome			
Epilepsy	~86% Europeans	15,212 cases/29,677 controls	ILAE consortium
Epilepsy	Europeans	4588 cases/144,780 controls	FinnGen consortium R4 release
Exposure			
Alcohol	Europeans	941,280 individuals	GWAS and Sequencing Consortium of Alcohol and Nicotine use (GSCAN)
Coffee	Europeans	375,833 individuals	UK biobank and US cohorts
Milk	Europeans	73,715 individuals	Copenhagen General Population Study

## Data Availability

The data used to conduct the analyses in the present study were obtained from public GWASs summary statistics (please see Supplementary Tables S1 and S2).

## Supplementary Materials

The following supporting information can be downloaded at: https://www.mdpi.com/article/10.3390/nu14061153/s1, Table S1: Characteristics of the SNPs associated with alcohol consumption; Table S2: Characteristics of the SNPs associated with coffee consumption.
