# Supplementary material for: Alcohol, Coffee, and Milk Intake in Relation to Epilepsy Risk
Source: Nutrients. 2022 Mar 9;14(6):1153. doi: 10.3390/nu14061153 (PMC8951548; doi:10.3390/nu14061153)
Supplement: Supplementary file 1 [file nutrients-14-01153-s001.zip › nutrients-1593859-supplementary.pdf]

Supplementary Table S1. Characteristics of the SNPs associated with alcohol consumption

SNP	Chr	effect_allele	other_allele	EAF	beta	se	p
rs705687	1	G	A	0.79	-0.0109	0.001776	8.15E-10
rs58107686	1	A	C	0.33	-0.00975	0.001585	7.79E-10
rs12088813	1	C	A	0.27	-0.00933	0.001649	1.58E-08
rs5024204	1	T	A	0.28	0.009703	0.001628	2.55E-09
rs10753661	1	A	G	0.68	-0.00864	0.001569	3.76E-08
rs28680958	1	A	G	0.22	-0.011	0.00177	5.13E-10
rs823114	1	A	G	0.55	0.008768	0.001467	2.31E-09
rs77165542	2	T	C	0.03	-0.02601	0.003971	5.63E-11
rs1260326	2	C	T	0.60	0.02089	0.001488	8.05E-45
rs13383034	2	T	C	0.33	0.014927	0.001551	6.31E-22
rs13032049	2	G	A	0.28	0.010195	0.001618	3E-10
rs828867	2	A	G	0.55	0.008757	0.001464	2.15E-09
rs11692435	2	A	G	0.09	0.01745	0.002616	2.53E-11
rs13024996	2	A	C	0.36	-0.01091	0.001515	5.72E-13
rs72859280	2	T	G	0.04	0.022885	0.003902	4.44E-09
rs56337305	2	C	T	0.38	-0.00959	0.001499	1.63E-10
rs13094887	3	T	A	0.30	-0.01031	0.001589	8.57E-11
rs62250685	3	G	A	0.61	-0.01436	0.0015	1.05E-21
rs13066454	3	T	C	0.40	-0.00878	0.001492	4.13E-09
rs9838144	3	C	G	0.21	-0.00996	0.001793	2.65E-08
rs2011092	3	C	T	0.34	-0.0089	0.00154	7.35E-09
rs6787172	3	G	T	0.55	-0.00803	0.001466	4.27E-08
rs3748034	4	T	G	0.14	-0.01174	0.002082	1.67E-08
rs11940694	4	G	A	0.60	0.02595	0.001486	3.03E-68
rs4501255	4	G	C	0.24	0.010693	0.001719	4.83E-10
rs1229984	4	C	T	0.96	0.150534	0.003861	<2.2e-308
rs36052336	4	G	A	0.06	-0.01843	0.003034	1.23E-09
rs2165670	4	A	G	0.11	0.02308	0.002364	1.67E-22
rs79139602	4	T	A	0.02	0.060272	0.005076	1.8E-32
rs4699791	4	A	G	0.10	0.018571	0.002477	6.58E-14
rs13107325	4	T	C	0.07	-0.0275	0.002816	1.53E-22
rs4690727	4	G	C	0.72	0.010817	0.00162	2.43E-11
rs12651313	4	G	C	0.44	-0.00864	0.001467	3.79E-09
rs4916723	5	C	A	0.42	-0.00995	0.001479	1.72E-11
rs12655091	5	A	G	0.53	-0.00831	0.00146	1.25E-08
rs55872084	5	T	G	0.24	0.009979	0.001719	6.32E-09
rs6460047	7	C	T	0.21	0.011624	0.001796	9.69E-11
rs10236149	7	G	A	0.12	-0.0135	0.002219	1.18E-09
rs35034355	7	A	G	0.52	-0.0081	0.001459	2.87E-08
rs6951574	7	C	T	0.46	0.013222	0.001463	1.58E-19
rs13250583	8	T	C	0.21	-0.00972	0.00178	4.7E-08
rs1217091	8	C	T	0.81	0.012161	0.001865	7.05E-11
rs28601761	8	G	C	0.42	0.009103	0.001477	7.17E-10
rs55932213	9	G	A	0.74	0.009488	0.001654	9.55E-09
rs10978550	9	C	T	0.21	-0.01175	0.001802	7.15E-11
rs7074871	10	A	G	0.26	-0.0094	0.001672	1.86E-08
rs17665139	10	T	C	0.15	-0.01156	0.002047	1.59E-08
rs7950166	11	T	C	0.64	-0.0098	0.001516	9.89E-11
rs11030084	11	T	C	0.18	-0.01061	0.001881	1.72E-08
rs56030824	11	A	G	0.32	-0.0116	0.001563	1.15E-13
rs10750025	11	T	C	0.69	0.010321	0.00157	4.89E-11

rs1713676	11	G	A	0.52	-0.00799	0.001459	4.29E-08
rs4938230	11	A	C	0.84	0.01281	0.001998	1.48E-10
rs682011	11	C	T	0.56	0.008212	0.001468	2.22E-08
rs12795042	11	C	A	0.62	-0.00832	0.001504	3.25E-08
rs10876188	12	T	C	0.46	-0.00799	0.001463	4.84E-08
rs3809162	12	G	A	0.40	0.009061	0.00149	1.19E-09
rs10506274	12	T	G	0.48	-0.00904	0.001458	5.78E-10
rs4842786	12	A	G	0.58	-0.0088	0.001479	2.73E-09
rs500321	13	T	A	0.74	-0.00967	0.001653	4.92E-09
rs1123285	14	G	C	0.34	-0.0089	0.001544	8.14E-09
rs2180870	14	C	T	0.14	-0.01218	0.002133	1.12E-08
rs28929474	14	T	C	0.02	-0.0368	0.005438	1.34E-11
rs11625650	14	A	G	0.23	-0.00957	0.001724	2.89E-08
rs2472297	15	T	C	0.25	0.010606	0.001685	3.1E-10
rs12907323	15	G	A	0.41	0.008497	0.001481	9.93E-09
rs2764771	16	A	G	0.31	0.009891	0.001582	4.02E-10
rs17177078	16	T	C	0.06	-0.02232	0.003012	1.27E-13
rs378421	16	A	G	0.40	-0.01121	0.001487	4.83E-14
rs11344371	16	A	G	0.31	-0.01021	0.001585	1.19E-10
rs62044525	16	G	C	0.18	-0.01217	0.001883	1.03E-10
rs7185555	16	C	G	0.15	-0.0111	0.002027	4.24E-08
rs79616692	16	C	G	0.11	0.016302	0.002351	4.11E-12
rs1104608	16	C	G	0.43	-0.01097	0.001476	1.05E-13
rs4548913	17	A	G	0.63	-0.00836	0.001511	3.11E-08
rs3803800	17	G	A	0.79	0.011379	0.001777	1.5E-10
rs2854334	17	G	A	0.62	0.009221	0.001498	7.51E-10
rs2532276	17	A	C	0.22	-0.0218	0.002559	1.62E-17
rs10438820	17	T	C	0.70	0.008972	0.001593	1.76E-08
rs9950000	18	T	C	0.40	-0.00912	0.001491	9.38E-10
rs4092465	18	G	A	0.64	-0.00829	0.001514	4.39E-08
rs281379	19	A	G	0.51	0.013722	0.001458	4.91E-21
rs4815364	20	A	G	0.62	0.008582	0.001499	1.02E-08
rs9607814	22	A	C	0.20	-0.01018	0.001859	4.31E-08

Supplementary Table S2. Characteristics of the SNPs associated with coffee consumption

SNP	Chr	effect_allele	other_allele	EAF	beta	se	p
rs574367	1	T	G	0.21	1.05	0.18	8.06E-09
rs10865548	2	G	A	0.83	1.54	0.19	4.46E-15
rs1260326	2	C	T	0.61	1.36	0.15	2.62E-19
rs1057868	7	T	C	0.29	1.97	0.16	5.26E-33
rs34060476	7	G	A	0.13	1.89	0.22	5.06E-18
rs4410790	7	C	T	0.63	3.94	0.15	5.59E-141
rs73073176	7	C	T	0.87	2.31	0.22	5.56E-25
rs597045	11	A	T	0.69	1.07	0.16	6.62E-11
rs1956218	14	G	A	0.56	0.82	0.15	3.62E-08
rs2472297	15	T	C	0.27	4.54	0.17	5.19E-155
rs66723169	18	A	C	0.23	1.47	0.18	9.88E-17
rs2330783	22	G	T	0.99	4.53	0.63	1.57E-12
